# A Comparative Study of Three Detection Techniques for* Leifsonia xyli* Subsp.* xyli*, the Causal Pathogen of Sugarcane Ratoon Stunting Disease

**DOI:** 10.1155/2018/2786458

**Published:** 2018-05-23

**Authors:** Qibin Wu, Yong-Bao Pan, Dinggang Zhou, Michael P. Grisham, Shiwu Gao, Yachun Su, Jinlong Guo, Liping Xu, Youxiong Que

**Affiliations:** ^1^Key Laboratory of Sugarcane Biology and Genetic Breeding, Fujian Agriculture and Forestry University, Ministry of Agriculture, Fuzhou 350002, China; ^2^USDA-ARS, Southeast Area, Sugarcane Research Unit, Houma, LA 70360, USA; ^3^Hunan University of Science and Technology, Xiangtan 411201, China

## Abstract

The ratoon stunting disease (RSD), caused by the bacterium* Leifsonia xyli *subsp*. xyli *(*Lxx*), is one of the most economically devastating diseases impacting sugarcane. RSD causes significant yield losses and variety degradation. Diagnosis of RSD is challenging because it does not exhibit any discernible internal and external symptoms. Moreover, the* Lxx* bacteria are very small and difficult to isolate, cultivate, and detect. In this study, conventional polymerase chain reaction (PCR), real-time quantitative PCR (RT-qPCR), and* Lxx*-loop-mediated isothermal amplification (*Lxx*-LAMP) were utilized to specifically detect the presence of* Lxx* pathogens in the juice from* Lxx*-infected sugarcane stalks and an* Lxx*-pMD18-T recombinant plasmid. The results showed that* Lxx* was a highly specific causal pathogen for RSD. All three techniques provided great reproducibility, while* Lxx*-LAMP had the highest sensitivity. When the DNA extract from* Lxx*-infected sugarcane juice was used as a template,* Lxx*-LAMP was 10 and 100 times more sensitive than RT-qPCR and conventional PCR, respectively. When the* Lxx*-pMD18-T recombinant plasmid was used as a template,* Lxx*-LAMP was as sensitive as RT-qPCR but was 10 times more sensitive than conventional PCR. Based on the* Lxx*-LAMP detection system established, adding 0.4 *μ*M loop primers (LF/LP) can accelerate the reaction and reduce the total time required. In addition, the optimal amount of* Bst* DNA polymerase for* Lxx*-LAMP reactions was determined to be 6.0 U. The results provide technical support for the detection of RSD* Lxx* pathogen that will help manage sugarcane RSD.

## 1. Introduction

The ratoon stunting disease (RSD), first detected in 1944–1945 from sugarcane cultivar Q 28 in Queensland, Australia, is now recognized as one of the most devastating sugarcane diseases worldwide [1]. The disease is caused by a bacterium that colonizes in the xylem vessels of the sugarcane plant. Davis et al. initially named the infectious agent* Clavibacter xyli* subsp.* xyli *(*Cxx*) based on the morphology of the bacterium [2]; however, Evtushenko et al. renamed it* Leifsonia xyli *subsp.* xyli *(*Lxx*) after evaluation of its rRNA gene characteristics [3]. Sugarcane plants with RSD infection usually show a reduction in stalk height (stunting), stalk diameter, and number of tillers. These symptoms may become worse as the perennial roots age. However, these symptoms are very similar to the stunted growth caused by drought or inefficient field management. As a result, diagnosis of RSD based on visual inspection is very difficult. As a result, transmission of the* Lxx* pathogen from field to field by propagating cuttings from infected plants is common [4]. RSD can cause yield losses of 12%–37% under normal conditions and up to 60% under drought conditions. Moreover, RSD may also lead to variety degradation [5–7].

The* Lxx* bacteria are very small and difficult to isolate, cultivate, and detect [8]. Current techniques for RSD diagnosis mainly include microscope inspection, serological tests, and DNA-based molecular detection. Damann discovered a host response to the presence of the causal bacterium in the metaxylem of sugarcane with RSD [9]. This response, alkaline-induced metaxylem autofluorescence (AIMA), can be used to detect* Lxx *under dark-field microscopy for RSD diagnosis. However, this method is not sensitive or accurate. Later, Roach and Hoy et al. detected RSD causal pathogen directly from sugarcane juice using phase contrast microscopy (PCM) [10, 11]. This method is more accurate than the AIMA method and can determine the number of pathogens quantitatively, but its sensitivity is still not satisfactory and the procedure is tedious and complicated. Enzyme-linked immunosorbent assay based techniques include dot blot enzyme immunoassays (DB-EIA) [12], evaporative-binding enzyme immunoassays (EB-EIA) [13], and tissue blot enzyme immunoassays (TB-EIA) [14]. In 1980, the successful isolation and cultivation of sugarcane* Lxx* bacteria enabled application of immunoassays [15]. Since 1984, many researchers have applied immunological techniques for the diagnosis of RSD. Matthews used ELISA for detection of the causal pathogen of sugarcane RSD and it was able to test 700 samples per day, while phase contrast microscopy can only test 50–100 samples per day [16]. Shen et al. compared the diagnostic accuracy of the internal symptoms inspection technique and DB-EIA and found that the former method was less reliable, while DB-EIA was more accurate and had higher sensitivity [17]. Later, Shen et al. utilized a DB-EIA assay and detected 28.4% RSD incidence from 232 sugarcane stalk samples randomly collected from the Weng-yuan sugarcane production region [18]. Li et al. (2010a) developed a simple, rapid, accurate, and effective TB-EIA assay for RSD detection that was suitable for high-throughput diagnosis in the field [19]. Hoy et al. compared the accuracy, false positive rate, and false negative rate of five diagnosis techniques including AIMA, microscopic inspection, DB-EIA, EB-EIA, and TB-EIA. The results demonstrated that TB-EIA provides the highest sensitivity and accuracy [11].

PCR is a more accurate method of detection over microscopic inspection and serological tests. Pan et al. pioneered the development of a PCR protocol for the specific detection of* Lxx*. Based on the intergenic transcribed spacer (ITS) region of the 16S–23S ribosomal DNA (NCBI nucleotide database number: AF056003), two* Lxx*-specific primers that amplified a 438 bp PCR product were designed [20]. In the same year, Fegan et al. reported another two* Lxx*-specific primers that amplified a 278 bp PCR product. Since then, these two sets of primers have been widely used to detect sugarcane RSD [21]. For example, Deng et al. reported PCR detection of RSD in sugarcane samples from Guangxi Province, China [22, 23]. Shen et al. detected an* Lxx* isolate from Guangdong Province of China that shared almost 100% nucleotide sequence identity with those from Australia, Brazil, and USA [24]. Dan et al. were able to detect* Lxx* through PCR in virus-free seeds of sugarcane [25]. Zhou et al. further improved the detection accuracy using nested-PCR [26]. Kazeem et al. conducted PCR analysis of DNA extracted from sugarcane sap of 76 cultivars in Nigeria. Although internal symptoms of RSD were observed in samples of cultivar Co 510, none of the sugarcane samples, including those from Co 510, yielded the 438 bp band expected for PCR detection of* Lxx *[27].

Real-time quantitative PCR (RT-qPCR) provides higher accuracy and sensitivity than conventional PCR [28–30]. As a result, RT-PCR is gaining increasing applications in the diagnosis and quantification of causal pathogen in plants [30–32]. In 2007, Grisham et al. developed an RT-PCR protocol for early* Lxx* detection in sugarcane. Because of its quantitative capability, real-time PCR was used to rank cultivars for susceptibility to* Lxx* infection [33].

In 2000, Notomi et al. reported a novel PCR technique known as loop-mediated isothermal amplification (LAMP) [34]. This technique employs a set of four specifically designed primers that recognize a total of six distinct sequences on the target gene. The reaction mixture contains a strand displacement DNA polymerase (*Bst*) and is kept under isothermal condition (65°C) for a period of time to obtain the final PCR product. The LAMP technique does not require heat denaturation of the template, thermal cycling, or gel electrophoresis of the final product. Instead, the amplified DNA product can be analyzed by staining of fluorescence dye or measuring of the turbidity of a byproduct, magnesium pyrophosphate. The LAMP technique is simple, quick, and highly specific. The technique has been utilized for the detection of genetically modified crops [35–38], as well as viruses [39–41], fungi [42], or bacteria [43–45] in the plants. In 2013, Liu et al. successfully developed an* Lxx-*LAMP protocol for the detection of* Lxx* in RSD-infected sugarcane. When the total DNA extracted from sugarcane juice was used as a template, LAMP detection of* Lxx* was 10 times more sensitive than conventional PCR [43]. Su et al. developed a LAMP protocol targeting the core effector* pep1 *gene of the sugarcane smut pathogen,* Sporisorium scitamineum*. Although the LAMP method was equally sensitive to conventional PCR in amplifying the* pep1* gene, it was 100 times more sensitive amplifying the* bE* gene of* S. scitamineum *[42].

In the present study, three molecular diagnostic techniques, namely, conventional PCR, RT-qPCR, and* Lxx*-LAMP, were used to specifically detect* Lxx* DNA in a dilution series of both DNA samples extracted from the juice of* Lxx*-infected stalks of sugarcane cultivar Yue-gan 18 and* Lxx*-pMD18-T recombinant plasmids. The sensitivities of these techniques in terms of the lowest detection limit of* Lxx* were determined. Loop primers and the amount of* Bst* DNA polymerase were optimized to improve the* Lxx*-LAMP technique. The results from this study will provide a scientific basis for selecting the best molecular diagnostic method to detect RSD infection in sugarcane.

## 2. Materials and Methods

### 2.1. Materials

Two sugarcane varieties, ROC 22 and Yue-gan 18 (also known as Guangdong sugar 00-236), were from the Key Laboratory of Sugarcane Biology and Genetic Breeding, Ministry of Agriculture, Fuzhou, Fujian Province, China. ROC 22 was free of RSD and negative for* Lxx*, while Yue-gan 18 was naturally* Lxx*-infected, from which* Lxx* bacteria were isolated. Two model bacteria,* Leifsonia ginseng* and* Leifsonia poae*, were purchased from the Agricultural Culture Collection of China (Beijing, China). The* Lxx*-pMD18-T recombinant plasmid was constructed by inserting the 438 bp Cxx1/Cxx2-PCR amplicon [20] into the pMD18-T Vector.

### 2.2. Methods

#### 2.2.1. Extraction of DNA from Sugarcane Juice

Material pretreatment: sugarcane xylem sap was collected into a 2.0 ml sterile centrifuge tube and centrifuged at 3,000 × g for 5 min at room temperature. The supernatant was then transferred to a new sterile centrifuge tube and centrifuged at 12,000 × g for 10 min at room temperature. The resulting supernatant was discarded and the pellet was kept.

The DNA was extracted from the pellets based on the CTAB method reported by Pan et al. with a minor modification [20]. The collected pellets were transferred into the cold mortar; after grinding the samples with liquid nitrogen, the ground samples were transferred into the 1.5 ml centrifuge tube. 1.0 ml CTAB extraction buffer (with 1*μ*L mercaptoethanol) was added and then incubated for about 60 min at 65°C, mixed occasionally by hand. 0.5 ml phenol: chloroform: isoamyl alcohol (25: 24: 1) was added to each sample; the tubes were gently inverted, rocked back and forth to mix well, and then centrifuged at 12,000 × g for 10 min at room temperature. 500*μ*L upper aqueous phase was transferred to a new 1.5 ml centrifuge tube; then, 500*μ*L cold isopropyl alcohol was added, mixed well by inverting the tube several times, and then incubated at -20°C for at least 2 hours. The DNA pellet was obtained by centrifuging at 12,000 × g and 4°C for 15 min, and then the supernatant was removed and washed twice with 500 *μ*L 75% ethanol. Following removal of the ethanol, the DNA pellet was centrifuged at 4,000 × g for 2 min at 4°C, after which the remaining ethanol was removed by pipette. The resultant DNA pellet was air-dried in a clean hood for about 20 min until transparent.

#### 2.2.2. Preparation of* Lxx*-Infected Sugarcane Juice and Plasmid DNA Samples

The DNA concentrations of* Lxx*-infected juice DNA and* Lxx*-pMD18-T recombinant plasmid were found to be 100 ng/*μ*L and 25 ng/*μ*L, respectively, using a NanoDrop spectrophotometer (Thermo Fisher Scientific, Waltham, MA, USA). After the initial concentration was determined, the two DNA samples were subjected to 10-fold serial dilutions. For* Lxx*-infected juice DNA sample, eight dilutions were prepared, namely, 10^0^, 10^−1^, 10^−2^, 10^−3^, 10^−4^, 10^−5^, 10^−6^, and 10^−7^. For* Lxx*-pMD18-T plasmid sample, twelve dilutions were prepared, 10^0^, 10^−1^, 10^−2^, 10^−3^, 10^−4^, 10^−5^, 10^−6^, 10^−7^, 10^−8^, 10^−9^, 10^10^, and 10^−11^. Next, 1.0 *μ*l from each dilution was subjected to PCR reaction. Sterile water was used as a blank control and DNA extracted from* Lxx*-free ROC 22 sugarcane juice was used as a negative control.

#### 2.2.3. Conventional PCR Detection of* Lxx*

The PCR was conducted on a Veriti 96-well thermal cycler (ABI, Foster City, CA, USA), and the reaction system and thermal cycles were according to Pan et al. with minor modification [20]. The PCR reaction mixture was composed of 2.5 *μ*L 10 × Ex Taq PCR buffer, 2.5 *μ*L 1.0% BSA, 2.0 *μ*L dNTPs (2.5 mM each), 0.5 *μ*L each of primers Cxx1 (10 *μ*M) and Cxx2 (10 *μ*M) (Table 1), 0.125 *μ*L Ex Taq polymerase (5 U/*μ*L), 1.0 *μ*L DNA template, and ddH_2_O to a final volume of 25 *μ*L. The thermal cycling program was 95°C for 10 min; 35 cycles (of 95°C for 30 s, 56°C for 30 s, and 72°C for 40 s); and 72°C for 5 min. PCR reactions were then held at 4°C until subsequent analysis. 5.0 *μ*L of the PCR product was separated by 1.5% agarose gel electrophoresis photographed using a multifunctional gel imaging system and subsequently analyzed.

#### 2.2.4. Real-Time Quantitative PCR

The RT-qPCR reaction mixture consisted of 12.5 *μ*L FastStart Universal SYBR Green Master (ROX) (Roche, Shanghai, China), 0.75 *μ*L each of Lxx82F (10 *μ*M) and Lxx22R (10 *μ*M) (Table 1), 1.0 *μ*L DNA template, and 10.0 *μ*L sterile ddH_2_O to give a final volume of 25 *μ*L.

The RT-qPCR program was conducted according to Grisham et al. with minor modification [33]. Briefly, samples were subjected to 95°C for 10 s, followed by 35 cycles of 94°C for 40 s, 64°C for 45 s, and a melting process composed of 94°C for 15 s, 64°C for 1 min, and 94°C for 15 s. Each sample was performed in triplicate at each repeat. RT-qPCR was conducted on an Applied Biosystems 7500 Real-Time PCR System (ABI, Foster City, CA, USA). At the end of the reaction, the Ct value for each dilution was analyzed.* Lxx* was considered to be present if a positive result was observed in less than 35 cycles.

The amplification efficiency of the Lxx82F/Lxx22R primer pair was determined using a real-time PCR standard curve that was represented as a semi-log regression line plot of Ct values versus (−log) of the input DNA template amount. The efficiency (E) of the real-time PCR assay was calculated using E = (10^−1/slope^) − 1. Theoretically, when 0.99 < R^2^ < 1.0, the slope of standard curve was considered valid. The results of RT-qPCR using the specific primer pair were validated when 0.9 < E < 1.1, with an E value closer to 1.0 indicating higher amplification efficiency.

#### 2.2.5. *Lxx*-LAMP Assay

The* Lxx*-LAMP reaction mixture was set up according to Liu et al. (2013). Briefly, the mixture consisted of 10 mM KCl, 20 mM Tris-HCl (pH 8.8), 10 mM (NH_4_)_2_SO_4_, 5.75 mM MgSO_4_, 0.1% Triton X-100, 0.2 *μ*M each of external primers F3 and B3, 0.8 *μ*M each of internal primers FIP and BIP (Table 1), 8 U* Bst* DNA polymerase, 1.4 mM dNTPs, and ddH_2_O to a final volume of 25 *μ*L. The mixture was incubated at 65°C for 60 min and then heated at 80°C for 3 min to terminate the reaction. LAMP products were kept at 4°C until further analysis by adding 1.0 *μ*L SYBR Green I (1,000X) (New England Biolabs, USA). Samples that turned green were considered to be* Lxx*-positive, while those that remained orange were considered to be* Lxx*-negative.

Two Loop primers, namely, LF and LP (Table 1), were designed according to the DNA sequence of* Lxx* reported by Pan et al. (1998) using the Primer Explorer 4.0 software (http://primerexplorer.jp/e/).

## 3. Results

### 3.1. Detection Specificity of the RSD Causal Agent* Lxx*

**Figure 1:**
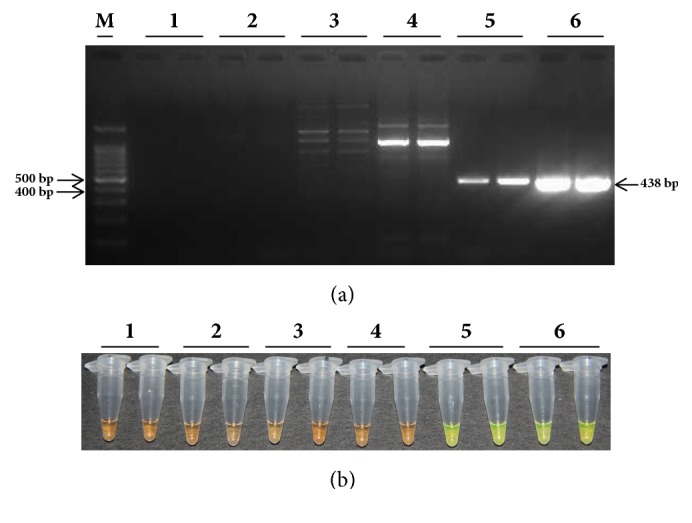
**The specificity of detecting* Leifsonia xyli* subsp.* xyli*, the causal pathogen of sugarcane ratoon disease.** (a) Agarose gel electropherograms of PCR amplification products. (b) Fluorescent color change of PCR amplification products. Lane M: 100 bp marker; Lane 1: ddH_2_O; Lane 2: 20 ng/*μ*L DNA sample extracted from* Lxx*-free sugarcane juice; Lane 3: 20 ng/*μ*L* Leifsonia poae* plasmid; Lane 4: 20 ng/*μ*L* Leifsonia ginsengi* plasmid; Lane 5: 20 ng/*μ*L DNA sample from* Lxx*-infected sugarcane juice; Lane 6: 20 ng/*μ*L* Lxx-*pMD18-T plasmid.

The RSD causal agent* Lxx* belongs to* Leifsonia *spp. In this study, two model strains of* Leifsonia* spp. were utilized, namely,* Leifsonia ginseng* and* Leifsonia poae*, to test the* Lxx* detection specificity by different methods. DNA samples prepared from xylem juice of RSD diseased Yue-gan 18 and recombinant plasmid* Lxx*-pMD18-T containing an* Lxx*-specific gene fragment were used as positive controls, while sterile water was used as a blank and DNA prepared from xylem juice of* Lxx*-free ROC 22 was used as a negative control. As shown in Figure 1(a), no* Lxx*-specific product was amplified from the blank and negative control. However, DNA sample from* Lxx*-infected xylem juice and recombinant* Lxx*-pMD18-T plasmid yielded a 438 bp* Lxx*-specific amplification product. In addition, no* Lxx*-specific product was amplified from* Leifsonia ginseng* and* Leifsonia poae.* Also shown in Figure 1(b), only the LAMP sample tubes containing* Lxx*-infected xylem juice DNA or recombinant* Lxx*-pMD18-T plasmid emitted green fluorescence. These results showed that the reaction mixtures were not contaminated and the RSD causal agent* Lxx* was highly specific to the molecular diagnostic techniques.

### 3.2. Detection of* Lxx* by Conventional PCR

**Figure 2:**
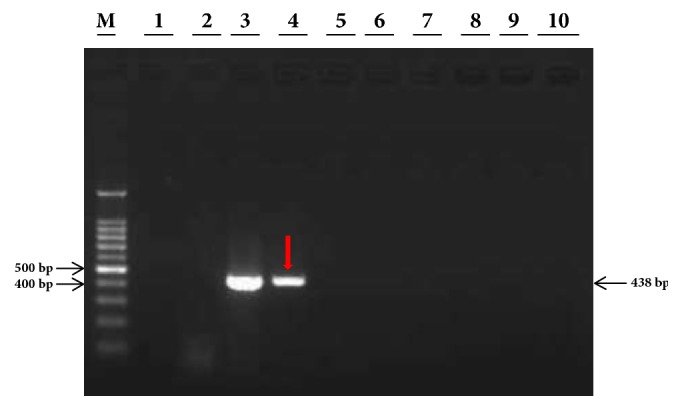
**Agarose gel electropherograms of conventional PCR products of DNA extract from* Lxx*-infected sugarcane juice.** Lane M: 100 bp molecular marker; Lane 1: ddH_2_O; Lane 2: 20 ng/*μ*L negative DNA extracted from* Lxx*-free juice; Lanes 3 to 10: 10-fold serial dilutions of DNA (10^0^ to 10^−7^, 4.0 ng/*μ*L to 4.0 × 10^−7^ ng/*μ*L) extracted from* Lxx*-infected sugarcane juice. The red arrow points to the limiting detection concentration of* Lxx*-infected sugarcane juice DNA.

**Figure 3:**
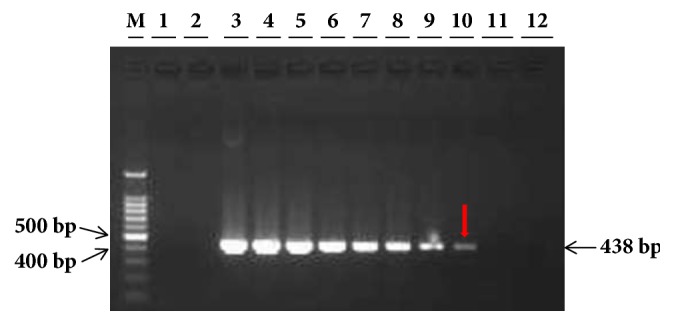
**Agarose gel electropherograms of conventional PCR products of* Lxx*-pMD18-T plasmids.** Lane M: 100 bp molecular marker; Lane 1: ddH_2_O; Lane 2: 20 ng/*μ*L negative DNA extracted from* Lxx*-free juice; Lanes 3 to 12: 10-fold serial dilutions of* Lxx-*pMD18-T plasmid (10^0^ to 10^−10^, 1.0 ng/*μ*L to 1.0 × 10^−10^ ng/*μ*L). The red arrow points to the limiting detection concentration of* Lxx-*pMD18-T plasmid.

The initial concentration of total DNA extracted from* Lxx*-infected juice of Yue-gan 18 and the* Lxx*-pMD18-T recombinant plasmid was 100 ng/*μ*L and 25 ng/*μ*L, respectively. Accordingly, in a 25 *μ*L reaction mixture, the concentration of the DNA template should be 4 ng/*μ*L and 1 ng/*μ*L for 10^0^ dilution, 0.4 ng/*μ*L and 0.1 ng/*μ*L for 10^−1^ dilution, and so on, for* Lxx*-infected juice DNA and* Lxx*-pMD18-T recombinant plasmid, respectively. In our study, the lowest level of* Lxx*-infected juice DNA detected by the conventional PCR using Cxx1/Cxx2 primers (Pan et al., 1998) was 0.4 ng/*μ*L (10^−1^ dilution) (Figure 2) and the lowest amount of* Lxx*-pMD18-T plasmid DNA by the conventional PCR using Cxx1/Cxx2 primers was 0.1 fg/*μ*L or 10^−7^ ng/*μ*L (10^−7^ dilution) (Figure 3).

### 3.3. Detection of* Lxx *by RT-qPCR

#### 3.3.1. Melt-Curve and Amplification Efficiency of the Lxx82F/Lxx22R Primers

**Figure 4:**
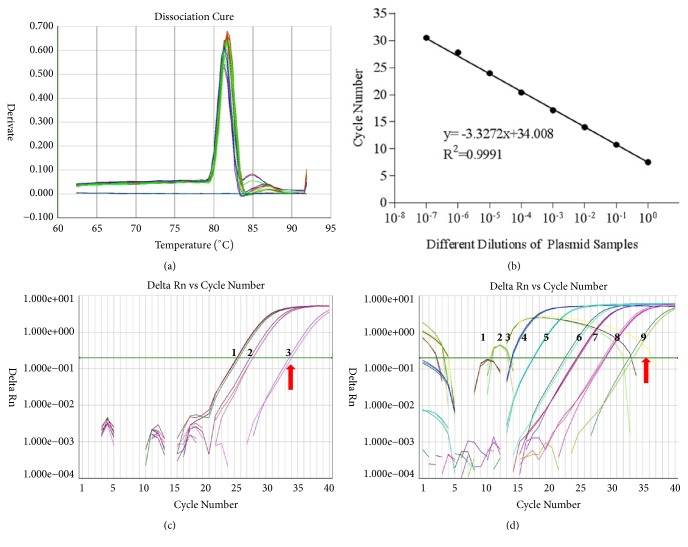
**Sensitivity assay of Lxx82F/Lxx22R primers set for* Lxx*-infected sugarcane juice DNA and* Lxx*-pMD18-T plasmid using RT-qPCR.** (a) Melt-curve analysis; (b) standard curve; (c) amplification plot of serial dilutions of DNA sample extracted from* Lxx*-infected sugarcane juice; templates 1-3 were 10-fold serial dilutions of* Lxx*-infected sugarcane juice DNA (10^0^ to 10^−2^, 4.0 ng/*μ*L to 0.04 ng/*μ*L); and (d) amplification plot of serial dilutions of* Lxx*-pMD18-T plasmid; templates 1-9 were 10-fold serial dilutions of* Lxx-*pMD18-T plasmid (10^0^ to 10^−8^, 1.0 ng/*μ*L to 1.0 × 10^−8^ ng/*μ*L). The red arrow points to the limiting detection concentration.

The melt-curve plots of amplification products using primer pair Lxx82F/Lxx22R are shown in Figure 4(a). A single peak melting profile representing a specific amplification product was observed, indicating that the Lxx82F/Lxx22R primers were highly specific to* Lxx* and could be used for the further detection of* Lxx*. As shown in Figure 4(b), the RT-qPCR amplification efficiency of the Lxx82F/Lxx22R primer pair was 1.01, which demonstrated that the primer pair was highly effective.

Each RT-qPCR amplification reaction was performed three times that showed reproducibility. The lowest amount of* Lxx*-infected juice DNA detected was 0.04 ng/*μ*L (10^−2^ dilution) (Table 2, Figure 4(c)) and the lowest limit of* Lxx*-pMD18-T plasmid detection was 10^−8^ ng/*μ*L (10^−8^ dilution) (Table 3, Figure 4(d))

### 3.4. Detection of* Lxx* by* Lxx*-LAMP

**Figure 5:**
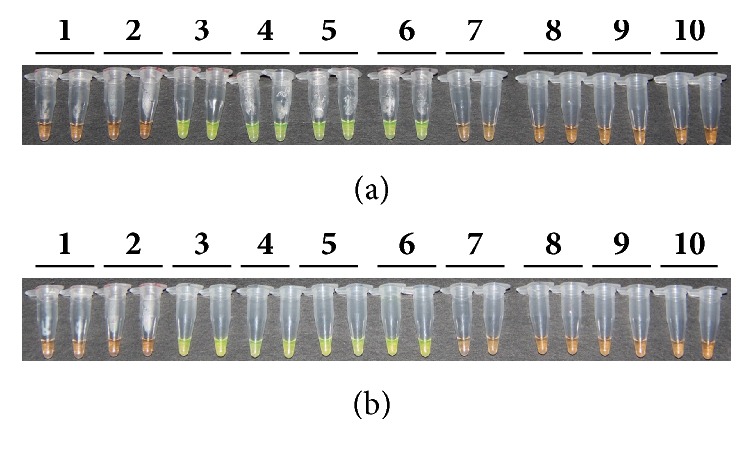
**Detection of products amplified by LAMP reactions with DNA extracted from* Lxx*-infected sugarcane juice based on color changes.** (a) Results of experiments conducted on 04/25/2013; (b) results of experiments conducted on 06/28/2013. Lane 1: ddH_2_O; Lane 2: 20 ng/*μ*L DNA sample extracted from* Lxx*-free sugarcane juice; Lanes 3 to 10: 10-fold serial dilutions of DNA (10^0^ to 10^−7^, 4.0 ng/*μ*L to 4.0 × 10^−7^ ng/*μ*L) extracted from* Lxx*-infected sugarcane juice.

**Figure 6:**
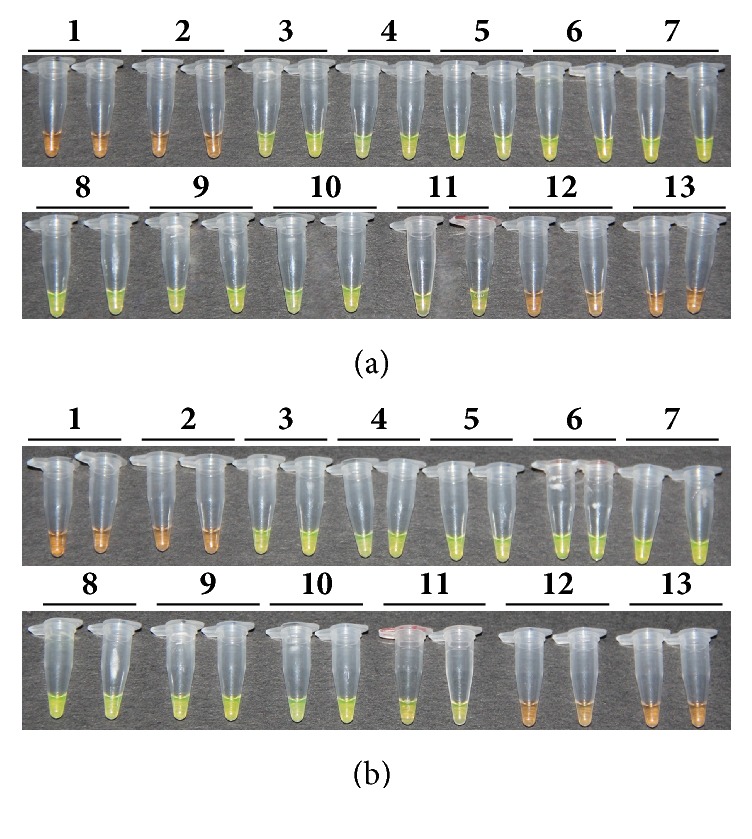
**Detection of products amplified by LAMP reactions with* Lxx*-pMD18-T plasmids.** (a) Results of experiments conducted on 04/25/2013; (b) results of experiments conducted on 06/28/2013. Lane 1: ddH_2_O; Lane 2: 20 ng/*μ*L negative plasmid DNA; Lanes 3 to 13: 10-fold serial dilutions of* Lxx-*pMD18-T plasmid (10^0^ to 10^−11^, 1.0 ng/*μ*L to 1.0 × 10^−11^ ng/*μ*L).

Each* Lxx*-LAMP amplification reaction was conducted in two tubes. The experiment was repeated on two different dates, one on 04/25/2013 and one on 06/28/2013. The amplification results were reproducible. The lowest detection limit was 0.004 ng/*μ*L (10^−3^ dilution) for* Lxx*-infected juice DNA (Figures 5(a) and 5(b)) and 10^−8^ ng/*μ*L (10^−8^ dilution) for* Lxx*-pMD18-T plasmid (Figures 6(a) and 6(b)).

### 3.5. Optimization of* Lxx*-LAMP Reaction Rate

**Figure 7:**
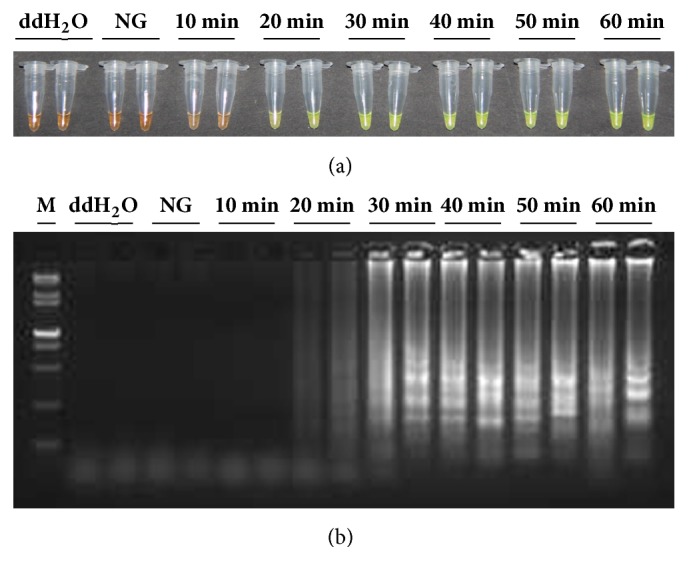
**Product amplified by* Lxx*-LAMP reaction in the presence of loop primers LF/LP.** (a) Detection of* Lxx*-LAMP products based on color change; (b) agarose gel electropherograms of* Lxx*-LAMP products. Lane M: 15,000 + 2,000 bp molecular marker; Lane NG: 20 ng/*μ*L* Lxx*-negative plasmid; Lanes 10 min to 60 min: the incubation time of LAMP reaction mixture containing 20 ng/*μ*L* Lxx*-positive plasmid.

To further reduce the reaction time of* Lxx*-LAMP, 0.4 *μ*M each of two additional loop primers, LF and LP, were added to an established* Lxx*-LAMP reaction mixture containing 20 ng* Lxx*-pMD18-T plasmid. The reaction system was then incubated at 65°C for 60 min; during this time an aliquot was taken out from the reaction system every 10 min and incubated at 80°C for 3 min to terminate the reaction. As shown in Figure 7(a), green fluorescence started to present after 20 min of incubation at 65°C. The results obtained from 2% agarose gel electrophoresis were similar, with ladder-like DNA bands starting to present after 20 min of incubation at 65°C (Figure 7(b)). These results demonstrated that addition of two more loop primers to the established* Lxx*-LAMP reaction mixture could accelerate the reaction rate.

### 3.6. Optimization of the Amount of* Bst* DNA Polymerase in* Lxx*-LAMP

**Figure 8:**
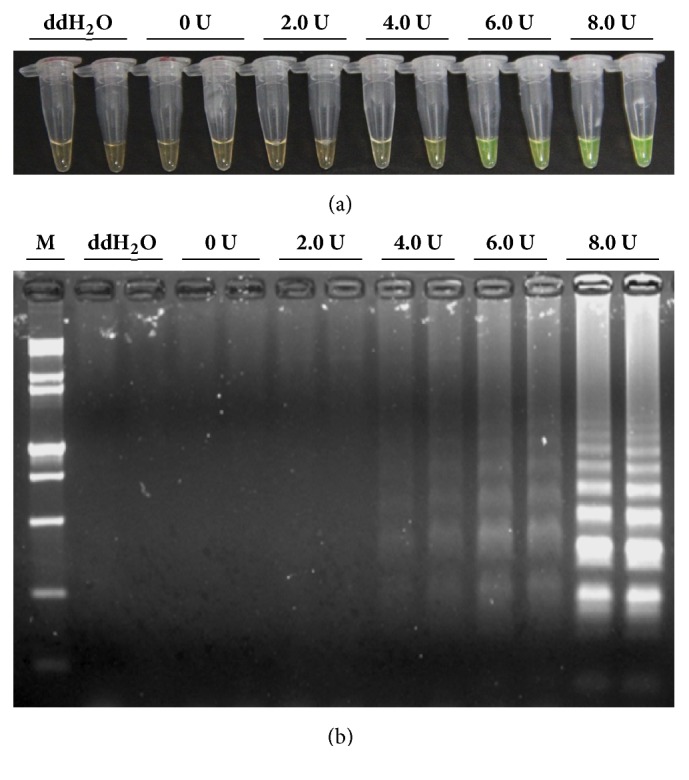
**Effects of different concentrations of* Bst* DNA polymerase on the LAMP reaction.** (a) Detection of* Lxx*-LAMP products based on color change; (b) agarose gel electropherograms of* Lxx*-LAMP products. Lane M: 15,000 + 2,000 bp molecular marker; Lanes 0 U to 8.0 U: LAMP reaction mixture containing 20 ng/*μ*L* Lxx*-positive plasmid plus 0 U, 2.0 U, 4.0 U, 6.0 U, and 8.0 U* Bst* DNA polymerase, respectively.

To find out the optimal amount of* Bst* DNA polymerase, five different enzyme concentrations, namely, 0 U, 2.0 U, 4.0 U, 6.0 U, and 8.0 U, were tested in the* Lxx*-LAMP reaction mixture (containing 20 ng/*μ*L* Lxx*-pMD18-T plasmid and 0.4 *μ*M loop primers). The reaction mixture was incubated at 65°C for 30 min, followed by 85°C for 5 min to terminate the reaction. The amplification product was analyzed by observing the color change upon SYBR Green I staining (Figure 8(a)) and 2% agarose gel electrophoresis (Figure 8(b)).

As shown in Figure 8(a), faint green fluorescence was observed at 4.0 U of the* Bst* DNA polymerase, while more intense green fluorescence was observed when 6.0 U or 8.0 U of the* Bst* DNA polymerase was used. The fluorescence intensity was readily detectable by the naked eye when 6.0 U of* Bst* DNA polymerase was included in the* Lxx-*LAMP reaction.

## 4. Discussion

RT-qPCR is a highly sensitive nucleic acid quantification technique based on PCR. This method is advantageous over conventional PCR because it offers higher accuracy, has tremendous sensitivity [28, 29], can be highly sequence-specific [46, 47], and requires simple yet rapid experimental procedures, little to no postamplification processing, and no agarose gel electrophoresis, making it less labor-intensive [48]. As a result, RT-qPCR has had increasing applications in the diagnosis and quantification of plant pathogens [30–32, 49]. Gao et al. utilized RT-qPCR for the diagnosis and quantification of* Fusarium solani f. sp. glycines* in soybean sudden death syndrome (SDS), which was the first report of using the comparative threshold cycle (Ct) method to quantify the DNA of a plant pathogen relative to its host DNA [31]. Liu et al. (2014) used multiplex PCR and SYBR Green real-time PCR to facilitate the simultaneous detection of three rice pathogens,* Xanthomonas oryzae* pv.* oryzae*,* Xanthomonas oryzae* pv.* oryzicola*, and* Burkholderia glumae *[49]. Kokkinos et al. developed an RT-qPCR protocol for diagnosis and quantification of the* sweet potato feathery mottle virus* (SPFMV),* sweet potato virus G* (SPVG),* Ipomoea vein mosaic virus* (IVMV),* Crinivirus sweet potato chlorotic stunt virus* (SPCSV), and the* Begomovirus sweet potato leaf curl virus* (SPLCV) directly from infected sweet potato plants. They found lower titers of SPFMV, IVMV, and SPVG in singly infected sweet potato plants than singly infected* Ipomoea setosa *Ker. and* I. nil *cv. Scarlet O'Hara plants. Kokkinos concluded that RT-qPCR was a more efficient method for detection of SPLCV than conventional PCR assay [30]. Sayler et al. successfully developed an RT-qPCR method that is effective for detecting and identifying the bacterium* Burkholderia glumae* in rice seed lots [32]. Grisham et al. developed an RT-qPCR assay to quantitatively detect the RSD causal pathogen* Leifsonia xyli *subsp*. xyli* (*Lxx*) from the sugarcane leaf tissue [33]. Because of its quantitative nature, RT-qPCR was used to rank cultivars for susceptibility to* Lxx* infection. In Grisham et al.'s study, two pairs of primers (Lxx202F/Lxx331R and Lxx82F/Lxx22R) that were suitable for* Lxx* detection were compared. Since Lxx82F/Lxx22R amplified nonspecific products, the Lxx202F/Lxx331R primer pair was considered optimal for* Lxx* amplification. In our study, however, Lxx82F/Lxx22R did not amplify nonspecific products and also showed higher amplification efficiency than Lxx202F/Lxx331R. We also demonstrated that the sensitivity of RT-qPCR was 10-fold higher than that of the conventional PCR method.

Currently, PCR and ELISA are widely used techniques for RSD diagnosis. However, PCR requires more expensive instruments and takes more than 2 h [4, 50]. ELISA also has some limitations, such as the requirement of a higher titer of* Lxx* pathogen in infected juice for detection [33]. Since the LAMP method was first described by Notomi et al. in 2000 [34], it has been widely applied in the diagnosis of bacterial and viral infection, as well as transgenic plant detection [37–45, 51]. Li et al. designed 5 primers targeting the* hly*A gene of* Listeria monocytogenes* (CMCC54001) and established a LAMP method for its detection [44]. Fukuta et al. developed immunocapture reverse transcription loop-mediated isothermal amplification (IC/RT-LAMP) for the detection of* tomato spotted wilt virus *(TSWV) [39]. This method enabled sensitive, reproducible, and specific detection of TSWV from chrysanthemum plants and was 100 times more sensitive than IC/RT-PCR. Liu et al. designed four specific primers targeting the* cp* gene of* tomato aspermy virus* (TAV), optimized reaction conditions, and established a LAMP method for TAV detection [41]. Their results demonstrated that the LAMP method was 1,000 times more sensitive than RT-PCR. Wang et al. investigated application of the LAMP method for detection of genetically modified crops. In their study, the* Cauliflower mosaic virus 35S* (*CaMV35S*) promoter gene, a widespread genetic element, was specifically amplified by the LAMP method [38]. Their results indicated that the LAMP method could detect a specific promoter containing* CaMV35S* and was 10 times more sensitive than conventional PCR.

Based on an* Lxx*-LAMP method developed by Liu et al. in 2013 [43], we added two additional loop primers to the* Lxx*-LAMP reaction system and reduced the total reaction time to 20–30 min. In addition, the presence or absence of target LAMP amplification products can be detected based on color change visible by the naked eye after SYBR Green I fluorescent dye staining. Moreover, when* Lxx*-infected juice DNA was used as a template, the* Lxx*-LAMP method was 10-fold and 100-fold more sensitive than RT-qPCR and conventional PCR, respectively. The* Lxx*-LAMP was 10-fold more sensitive than conventional PCR when* Lxx*-pMD18-T plasmid was used as a template. These results were consistent with those reported by Liu et al. [43], who found that the* Lxx*-LAMP method was 10-fold more sensitive than conventional PCR when* Lxx*^*+*^ juice DNA was used as a template.

In this study, three techniques, conventional PCR,* Lxx*-LAMP, and RT-qPCR, were used to detect the RSD casual pathogen* Lxx*. RSD is specifically caused by* Lxx* infection, which was first confirmed using the two nonpathogenic model bacteria of* Leifsonia *subsp. When* Lxx*-infected juice DNA was used as a template, the lowest DNA concentration that could be detected by conventional PCR,* Lxx-*LAMP, or RT-qPCR was 0.4 ng/*μ*L, 0.004 ng/*μ*L, and 0.04 ng/*μ*L, respectively. The results from the three different techniques conducted on different dates were consistent, demonstrating that all three techniques provided satisfactory reproducibility. The* Lxx*-LAMP method offered the highest sensitivity, being 10- and 100-folder higher than RT-qPCR and conventional PCR, respectively. When* Lxx*-pMD18-T was used as a template, the lowest DNA concentration that could be detected by conventional PCR was 10^−7^ ng/*μ*L, while for* Lxx*-LAMP and RT-qPCR they were both 10^−8^ ng/*μ*L and the results were reproducible in experiments conducted on different dates. These results demonstrated that the sensitivity of* Lxx*-LAMP and that of RT-qPCR are comparable, but that both are 10-fold higher than conventional PCR.

## 5. Conclusions

Conventional PCR,* Lxx*-LAMP, and RT-qPCR all provide reproducible results for its detection, with* Lxx*-LAMP being the most sensitive technique to detect* Lxx*. In addition, when two additional loop primers were added to the* Lxx*-LAMP reaction mixture, the reaction was accelerated and the reaction time reduced. Moreover, the optimal amount of* Bst* DNA polymerase large fragment was found to be 6.0 U when taking the cost and the feasibility of detecting the color change by the naked eye into account.

## Figures and Tables

**Table 1:** Sequences of PCR, LAMP, and RT-qPCR primers used for *Lxx* detection.

Primer	Sequence (5′to 3′)
Cxx1^a^	CCGAAGTGAGCAGATTGACC
Cxx2	ACCCTGTGTTGTTTTCAACG
F3^b^	ACATCGGTACGACTGGGT
B3	TGGCCGACCAAAAAAGGT
FIP	GGCGTACTAAGTTCGAGCCGTT-GGTCAGCTCATGGGTGGA
BIP	CCTCGCACATGCACGCTGTT-CTCAGCGTCTTGAAGACACA
LF	CTCCGCACCAATGTCAATGT
LP	CTGAGGGACCGGACCTCATC
Lxx82F^c^	TTCAACGCAGAAATTGTCCAGG
Lxx22R	CAAGCAGGCGTACTAAGTTCGA

^a^The Cxx1/Cxx2 primers were according to Pan et al. [20].

^b^The F3/B3 & FIP/BIP primers were according to Liu et al. [43].

^c^The Lxx82F/Lxx22R primers were according to Grisham et al. [33].

**Table 2:** Ct values of RT-qPCR using serial dilutions of DNA extracted from *Lxx*-infected sugarcane juice as templates.

Sample	2013.5.6^a^		2013.6.26^b^	
	Ct^c^	Mean Ct	Ct	Mean Ct
Black (ddH_2_O)	— ^d^	—	—	—
Negative juice DNA	—	—	—	—
10^0^ positive juice DNA	25.6029; 25.1696; 25.139	25.304	24.6533; 23.8346; 24.3586	24.2822
10^−1^ positive juice DNA	29.1754; 28.7072; 28.249	28.711	27.7062; 27.4855; 27.4397	27.5438
10^−2^ positive juice DNA	32.437; 32.138; 32.4321	32.336	31.3557; 32.5417; —	31.9487
10^−3^ positive juice DNA	—	—	—	—
10^−4^ positive juice DNA	—	—	—	—
10^−5^ positive juice DNA	—	—	—	—
10^−6^ positive juice DNA	—	—	—	—
10^−7^ positive juice DNA	—	—	—	—

^a^  Results of experiments conducted on 04/25/2013.

^b^  Results of experiments conducted on 06/26/2013.

^c^  “Ct” means cycle threshold.

^d^  “—” means absence detection of *Lxx*.

**Table 3:** Ct values of RT-qPCR using serial dilution of *Lxx*-pMD18-T plasmid as templates.

Sample	2013.5.6^a^		2013.6.26^b^	
	Ct^c^	Mean Ct	Ct	Mean Ct
Blank (ddH_2_O)	— ^d^	—	—	—
Negative plasmid	—	—	—	—
10^0^ *Lxx*-pMD18-T plasmid	8.3205; 8.4208; 8.2091	8.3168	8.5207; 7.8205; 7.947	8.096
10^−1^ *Lxx*-pMD18-T plasmid	11.5595; 11.4471; 11.3005	11.4357	11.2019; 11.4752; 10.9927	11.223
10^−2^ *Lxx*-pMD18-T plasmid	13.515; 13.4772; 13.5749	13.5224	13.62; 13.7713; 13.5248	13.639
10^−3^ *Lxx*-pMD18-T plasmid	14.7635; 14.6707; 14.6183	14.6842	16.1848; 16.1488; 16.2099	16.121
10^−4^ *Lxx*-pMD18-T plasmid	18.0854; 18.1271; 18.0325	18.0817	20.161; 20.0562; 19.7764	19.998
10^−5^ *Lxx*-pMD18-T plasmid	23.0183; 23.3196; —	23.169	24.3614; 24.4379; 23.9269	24.242
10^−6^ *Lxx*-pMD18-T plasmid	25.0384; 24.8664; 24.7652	24.8900	28.0877; 28.1149; 27.6601	27.955
10^−7^ *Lxx*-pMD18-T plasmid	29.1153; 19.8728; 29.2228	29.0703	30.9799; 30.3203; —	30.6501
10^−8^ *Lxx*-pMD18-T plasmid	33.412; 32.8918; —	33.1519	34.906; 32.892; 33.412	33.737
10^−9^ *Lxx*-pMD18-T plasmid	—	—	—	—
10^−10^ *Lxx*-pMD18-T plasmid	—	—	—	—

^a^  Results of experiments conducted on 04/25/2013.

^b^  Results of experiments conducted on 06/26/2013.

^c^  “Ct” means cycle threshold.

^d^  “—” means absence detection of *Lxx*.

## Data Availability

The data supporting the conclusions of this article are all within the paper.
